# Supplementary material for: Hybrid Models and Biological Model Reduction with PyDSTool
Source: PLoS Comput Biol. 2012 Aug 9;8(8):e1002628. doi: 10.1371/journal.pcbi.1002628 (PMC3415397; doi:10.1371/journal.pcbi.1002628)
Supplement: Text S4 — Complete source code for the PyDSTool package (version 0.88.120504). Includes API documentation and help files linking to web pages. This file is identical to the current public release on Sourceforge.net. (ZIP) [file pcbi.1002628.s004.zip › PyDSTool/html/PyDSTool.PyCont.ContClass'.ContClass-class.html]

xml version="1.0" encoding="ascii"?


PyDSTool.PyCont.ContClass'.ContClass


| Home | Trees | Indices | Help | PyDSTool |
| --- | --- | --- | --- | --- |

Package PyDSTool ::
Package PyCont ::
Module ContClass' ::
Class ContClass

[hide private]

[frames] | no frames]

# Class ContClass

source code

```
    object --+    
             |    
common.Utility --+
                 |
                ContClass
```

---

Stores continuation curves for a specified model.


|  |  |
| --- | --- |
| Instance Methods | [hide private] |

|  |  |
| --- | --- |
| \_\_contains\_\_(self, name) | source code |

|  |  |
| --- | --- |
| \_\_copy\_\_(self) | source code |

|  |  |
| --- | --- |
| \_\_deepcopy\_\_(self, memo=None, \_nil=`[``]`) | source code |

|  |  |
| --- | --- |
| \_\_getitem\_\_(self, name) | source code |

|  |  |
| --- | --- |
| \_\_init\_\_(self, model)  x.\_\_init\_\_(...) initializes x; see x.\_\_class\_\_.\_\_doc\_\_ for signature | source code |

|  |  |
| --- | --- |
| \_\_repr\_\_(self)  str(x) | source code |

|  |  |
| --- | --- |
| \_\_str\_\_(self)  str(x) | source code |

|  |  |
| --- | --- |
| compileAutoLib(self, libsources=`[``]`, libdirs=`[``]`)  compileAutoLib generates a python extension DLL with continuer and vector field compiled and linked. | source code |

|  |  |
| --- | --- |
| computeEigen(self) | source code |

|  |  |
| --- | --- |
| delCurve(self, curvename) | source code |

|  |  |
| --- | --- |
| display(self, coords=None, curves=None, figure=None, axes=None, stability=False, domain=False, \*\*plot\_args)  Plot all curves in coordinates specified by coords. | source code |

|  |  |
| --- | --- |
| exportGeomview(self, coords=None, filename=`'``geom.dat``'`) | source code |

|  |  |
| --- | --- |
| exportMatlab(self, filename=None) | source code |

|  |  |
| --- | --- |
| forceAutoLibRefresh(self)  forceAutoLibRefresh should be called after event contents are changed, or alterations are made to the right-hand side of the ODEs. | source code |

|  |  |
| --- | --- |
| info(self) | source code |

|  |  |
| --- | --- |
| loadAutoMod(self) | source code |

|  |  |
| --- | --- |
| makeAutoLib(self, libsources=`[``]`, libdirs=`[``]`, include=`[``]`)  makeAutoLib calls makeAutoLibSource and then the compileAutoLib method. | source code |

|  |  |
| --- | --- |
| makeAutoLibSource(self, include=`[``]`)  makeAutoLibSource generates the C source for the vector field specification. | source code |

|  |  |
| --- | --- |
| newCurve(self, initargs)  Create new curve with arguments specified in the dictionary initargs. | source code |

|  |  |
| --- | --- |
| update(self, args)  Update parameters for all curves. | source code |

**Inherited from `object`**:
`__delattr__`,
`__getattribute__`,
`__hash__`,
`__new__`,
`__reduce__`,
`__reduce_ex__`,
`__setattr__`


|  |  |
| --- | --- |
| Class Variables | [hide private] |

auto\_list = `['LC-C']`

curve\_args\_list = `['verbosity']`

curve\_list = `{'EP-C': <class 'PyDSTool.PyCont.Continuation.Equ...`


|  |  |
| --- | --- |
| Properties | [hide private] |

**Inherited from `object`**:
`__class__`


|  |  |
| --- | --- |
| Method Details | [hide private] |

|  |  |
| --- | --- |
| \_\_init\_\_(self, model)  *(Constructor)* | source code |

x.\_\_init\_\_(...) initializes x; see x.\_\_class\_\_.\_\_doc\_\_ for
signature

Overrides: object.\_\_init\_\_: *(inherited documentation)*

|  |  |
| --- | --- |
| \_\_repr\_\_(self)  *(Representation operator)* | source code |

str(x)

Overrides: object.\_\_repr\_\_: *(inherited documentation)*

|  |  |
| --- | --- |
| \_\_str\_\_(self)  *(Informal representation operator)* | source code |

str(x)

Overrides: object.\_\_str\_\_: *(inherited documentation)*

|  |  |
| --- | --- |
| compileAutoLib(self, libsources=`[``]`, libdirs=`[``]`) | source code |

```
compileAutoLib generates a python extension DLL with continuer and vector
field compiled and linked.

libsources list allows additional library sources to be linked.
libdirs list allows additional directories to be searched for
  precompiled libraries.
```

|  |  |
| --- | --- |
| display(self, coords=None, curves=None, figure=None, axes=None, stability=False, domain=False, \*\*plot\_args) | source code |

```
Plot all curves in coordinates specified by coords.

Inputs:

    coords -- tuple of coordinates (None defaults to the first free
        parameter and the first state variable)
```

|  |  |
| --- | --- |
| forceAutoLibRefresh(self) | source code |

forceAutoLibRefresh should be called after event contents are changed,
or alterations are made to the right-hand side of the ODEs.

Currently this function does NOT work!

|  |  |
| --- | --- |
| makeAutoLib(self, libsources=`[``]`, libdirs=`[``]`, include=`[``]`) | source code |

makeAutoLib calls makeAutoLibSource and then the compileAutoLib
method. To postpone compilation of the source to a DLL, call
makeAutoLibSource() separately.

|  |  |
| --- | --- |
| makeAutoLibSource(self, include=`[``]`) | source code |

makeAutoLibSource generates the C source for the vector field
specification. It should be called only once per vector field.

  


|  |  |
| --- | --- |
| Class Variable Details | [hide private] |

### curve\_list

Value:
:   ```
    {'EP-C': <class 'PyDSTool.PyCont.Continuation.EquilibriumCurve'>,
     'FP-C': <class 'PyDSTool.PyCont.Continuation.FixedPointCurve'>,
     'H-C1': <class 'PyDSTool.PyCont.Continuation.HopfCurveOne'>,
     'H-C2': <class 'PyDSTool.PyCont.Continuation.HopfCurveTwo'>,
     'LC-C': <class 'PyDSTool.PyCont.Continuation.LimitCycleCurve'>,
     'LP-C': <class 'PyDSTool.PyCont.Continuation.FoldCurve'>,
     'UD-C': <class 'PyDSTool.PyCont.Continuation.UserDefinedCurve'>}
    ```

  


| Home | Trees | Indices | Help | PyDSTool |
| --- | --- | --- | --- | --- |

|  |  |
| --- | --- |
| Generated by Epydoc 3.0.1 on Fri May 4 15:24:07 2012 | http://epydoc.sourceforge.net |
